# The HOS1-PIF4/5 module controls callus formation in *Arabidopsis* leaf explants

**DOI:** 10.1080/15592324.2023.2261744

**Published:** 2023-09-25

**Authors:** Kyounghee Lee, Dohee Koo, Ok-Sun Park, Pil Joon Seo

**Affiliations:** a Department of Chemistry, Seoul National University, Seoul, Korea; b Research Institute of Basic Sciences, Seoul National University, Seoul, Korea; c Plant Genomics and Breeding Institute, Seoul National University, Seoul, Korea

**Keywords:** Callus formation, HOS1, PIF4/5, auxin signaling, plant regeneration

## Abstract

A two-step plant regeneration has been widely exploited to genetic manipulation and genome engineering in plants. Despite technical importance, understanding of molecular mechanism underlying *in vitro* plant regeneration remains to be fully elucidated. Here, we found that the HIGH EXPRESSION OF OSMOTICALLY RESPONSIVE GENES 1 (HOS1)-PHYTOCHROME INTERACTING FACTOR 4/5 (PIF4/5) module participates in callus formation. Consistent with the repressive role of HOS1 in PIF transcriptional activation activity, *hos1–3* mutant leaf explants exhibited enhanced callus formation, whereas *pif4–101 pif5–3* mutant leaf explants showed reduced callus size. The HOS1-PIF4/5 function would be largely dependent on auxin biosynthesis and signaling, which are essential for callus initiation and proliferation. Our findings suggest that the HOS1-PIF4/5 module plays a pivotal role in auxin-dependent callus formation in *Arabidopsis*.

Some plant cells can form a mass of pluripotent cells, called callus. This not only occurs spontaneously at wound sites but can also be induced by *in vitro* tissue cultures. Callus can undergo *de novo* organ formation or embryogenesis, giving rise to a new organ or even an entire plant.^1–3^ During *in vitro* tissue culture, a concentration ratio of two phytohormones, auxin and cytokinin, determines cell fate transitions: incubation of tissue explants on auxin-rich callus-inducing medium (CIM) facilitates callus proliferation,^3,4^ whereas *de novo* shoot regeneration can be stimulated by incubation on the cytokinin-rich shoot-inducing medium (SIM).^3^

A particular emphasis has been placed on the callus formation process, because active callus proliferation facilitates pluripotency acquisition, which is a fundamental basis of plant regeneration.^5^ Accumulating evidence has shown that the CIM-derived callus resembles root primordium, regardless of origin of tissue explants.^6^ In *Arabidopsis*, callus formation is initiated from the pericycle-like cells,^4,7^ which then undergo asymmetric cell division to establish root primordium identity with the activation of auxin-inducible root developmental genes, such as *AUXIN RESPONSE FACTOR*s (*ARF*s) and *LATERAL ORGAN BOUNDARIES DOMAIN*s (*LBD*s).^8^ After acquisition of root primordium characteristics, callus cells establish a regeneration competence via expression of root stem cell regulators, including *PLETHORA*s (*PLT*s), *SCARECROW* (*SCR*), *WUSCHEL-RELATED HOMEOBOX 5* (*WOX5*), *WOX7*, and *WOX14*.^6,7,9^ Accordingly, *plt3 plt5 plt7*, *scr*, and *wox5 wox7 wox14* mutants exhibit impaired *de novo* shoot regeneration due to the failure of pluripotency acquisition.^9,10^

The HOS1 protein is involved in diverse aspects of plant growth and development, such as circadian clock, flowering, thermotolerance, and light and hormone signaling.^11–15^ In particular, the HOS1 protein is important for controlling auxin biosynthesis and signaling. The *hos1* mutants exhibited elongated hypocotyls possibly with increased auxin biosynthesis compared to wild-type in light condition.^12^ In addition, HOS1 also inhibits the transcriptional activation activity of PIF4 to further influence auxin signaling-related genes.^15^

Although plant tissue culture has been widely exploited to genome engineering of various plant species, its application is still limited in many plant species. Several lines of evidence have shown that a low regeneration capacity is frequently caused by genetic barriers that intrinsically block key steps of plant regeneration.^16–18^ To find out genetic obstacles for efficient plant regeneration, we screened several genetic mutants and tried to find mutants displaying enhanced callus formation and/or shoot regeneration. Our initial screening suggested that a *hos1* mutant showed an enhanced callus formation compared to wild type. To validate our preliminary results, we obtained two *HOS1*-deficient mutant alleles and examined callus formation rate. As expected, callus formation was significantly increased in *hos1* mutants compared to wild type, especially at a low concentration of exogenous auxin in CIM (Figure 1a).

**Figure 1.**
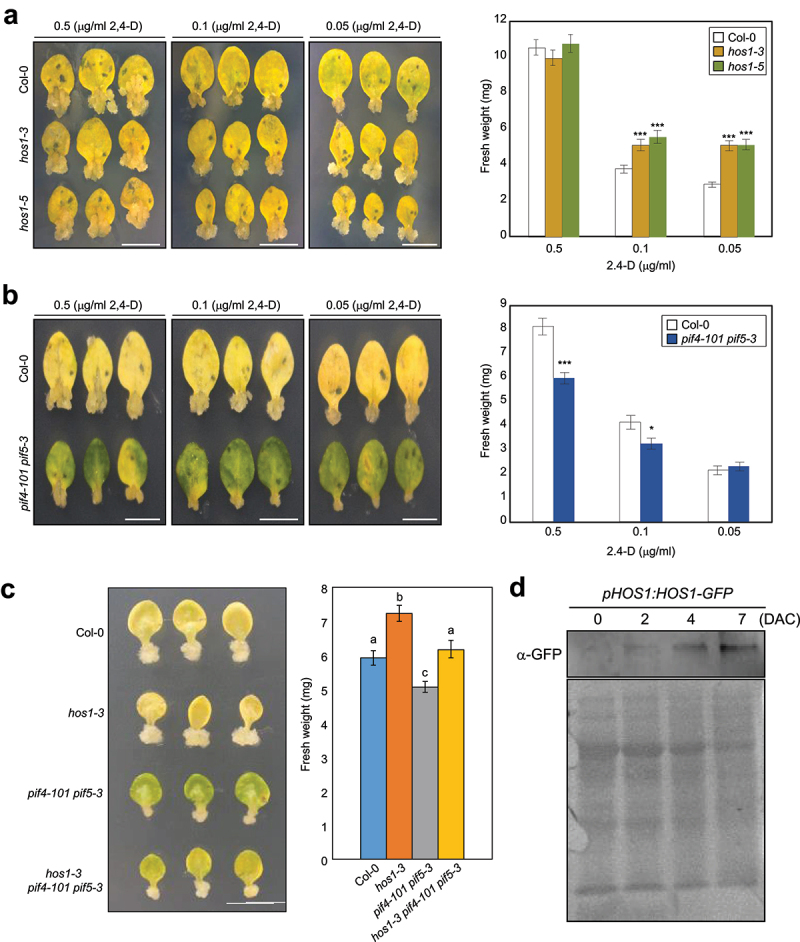
The HOS1-PIF4/5 module regulates callus formation.

(a) Role of HOS1 in callus formation. (b) Reduced callus formation in *pif4-101 pif5-3* mutant leaf explants. In (a) and (b), the third and fourth leaves of 2-week-old seedlings were used to induce calli on callus-inducing medium (CIM) supplemented with different concentrations of 2,4-D. Plates were incubated for 2 weeks under continuous dark conditions and photographed (left panels). Thirty calli excised from leaf explants were collected to measure fresh weight (right panels). Data represent the mean ± SEM. Asterisks indicate statistically significant differences (Student’s *t*-test, *P < 0.05; ***P < 0.001). Bars indicate the standard error of the mean. Scale bars = 5 mm. (c) Genetic analysis. Leaf explants from the third and fourth leaves of 2-week-old seedlings were used to induce calli on CIM supplemented with 0.1 µg/mL 2,4-D. Plates were incubated for 2 weeks under continuous dark conditions. Thirty calli excised from leaf explants were collected to measure fresh weight. Data represent the mean ± SEM. Statistically significant differences were determined using one-way analysis of variance (ANOVA), followed by Fisher’s *post hoc* test. Different letters indicate significant differences (*P < 0.05). Scale bars = 5 mm. (d) Accumulation of HOS1 during callus formation. Leaf explants from the third and fourth leaves of 2-week-old seedlings were used to induce calli on CIM. Leaf explants of *pHOS1:HOS1-eGFP* were incubated on CIM supplemented with 0.1 µg/mL 2,4-D for up to 7 d. Whole leaf explants with developing calli were collected for immunoblot analysis. The HOS1-eGFP protein was detected immunologically using an anti-GFP antibody. Coomassie blue-stained gel is shown as a loading control. DAC, days after incubation on CIM.

We next asked how HOS1 regulates callus formation. Since auxin signaling is closely associated with callus formation,^19–21^ we suspected that HOS1 might affect auxin biosynthesis and/or signaling. Interestingly, HOS1 is known to inhibit the transcriptional activation activity of PIF4, key regulator of auxin biosynthesis and signaling, without affecting its transcript accumulation.^15^ Consistent with this, an auxin-responsive gene regulated by PIF4/5 was upregulated in *hos1* mutants (Supplemental Figure S1). We thus examined whether *PIF4* and its redundant gene *PIF5* are indeed involved in callus formation. The phenotypic analysis showed that the *pif4–101 pif5–3* mutant exhibited smaller callus size than wild type (Figure 1b). To confirm the genetic hierarchy, we checked callus proliferation activity of *hos1–3 pif4–101 pif5–3* triple mutant. As a result, the enhanced callus size phenotype of *hos1–3* leaf explants was compromised in part by introducing *pif4–101 pif5–3* mutations (Figure 1c). These results indicate that the HOS1-PIF4/5 module plays an important role in regulating callus formation in *Arabidopsis*. Although HOS1 is likely dependent on PIF4 and PIF5 in the control of callus proliferation, we cannot rule out the possibility that additional biological functions of HOS1, such as ethylene signaling and circadian control,^14,22^ could also be linked to plant regeneration.^23,24^ Furthermore, considering the enhanced greening phenotype in *pif4–101 pif5–3* mutant leaf explants (Figure 1b), there might be additional developmental impact of the HOS1-PIF4/5 module in leaf senescence and/or cytokinin signaling control, which can influence *in vitro* plant regeneration.

Given that the HOS1 protein accumulated during callus proliferation (Figure 1d), this protein might constitute a negative feedback pathway of auxin biosynthesis and/or signaling that allows proper callus division rate during *in vitro* tissue culture. In this context, HOS1 may act as a genetic barrier that limits callus initiation as well as callus proliferation by restricting auxin biosynthesis and signaling. Since HOS1 is widely conserved across various plant species,^25^ inactivation of HOS1 may contribute to enhancing callus formation especially in crop and woody plants that have a low capability of callus formation.

## Materials and methods

### Plant materials and growth conditions

*Arabidopsis thaliana* ecotype Columbia (Col-0) was used for all experiments. Plants were grown at 22–23°C under long-day (LD) conditions (16 h light/8 h dark) using white fluorescent lamps (120 µmol photons m^−2^s^−1^). The *hos1–3* (SALK_069312), *hos1–5* (SAIL_1211_D02), and *pif4–101 pif5–3* mutants have been described previously.^26,27^ To induce callus formation, most recently emerging leaves (3^rd^ and 4^th^ leaves) obtained from 2-week-old plants were excised and placed on callus-inducing medium (CIM) [B5 medium supplemented with 0.05 µg/ml kinetin and 0.5 µg/ml 2, 4-dichlorophenoxyacetic acid [2,4-D] (or 0.1 µg/ml 2,4-D or 0.05 µg/ml 2,4-D)] and incubated at 22–23°C in the dark.

### Immunoblot analysis

Harvested plant materials were ground in liquid nitrogen, and total cellular extracts were suspended in sodium dodecyl sulfate-polyacrylamide gel electrophoresis (SDS-PAGE) sample loading buffer. Protein samples were analyzed using SDS-PAGE (10% gels) and blotted onto Hybond-P+ membranes (Amersham-Pharmacia). Proteins were immunologically detected using anti-GFP antibody (Abcam, ab290).

## Supplementary Material

Supplemental Material
