# Supplementary material for: The HOS1-PIF4/5 module controls callus formation in Arabidopsis leaf explants
Source: Plant Signal Behav. 2023 Sep 25;18(1):2261744. doi: 10.1080/15592324.2023.2261744 (PMC10761175; doi:10.1080/15592324.2023.2261744)
Supplement: Supplemental Material [file KPSB_A_2261744_SM5645.ppt]

## Slide 1
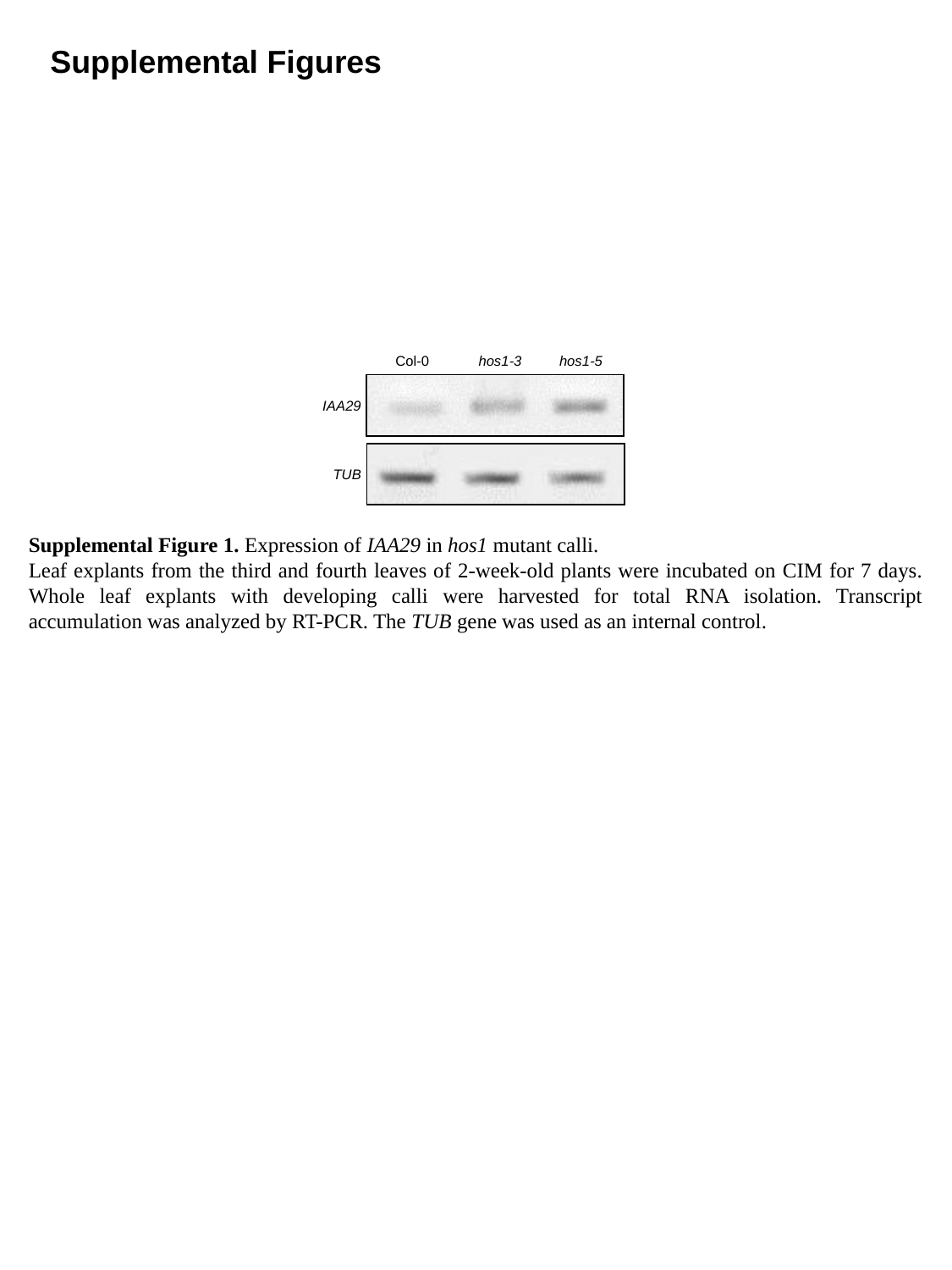

Supplemental Figures
Col-0
hos1-3
hos1-5
IAA29
TUB
Supplemental Figure 1. Expression of IAA29 in hos1 mutant calli.
Leaf explants from the third and fourth leaves of 2-week-old plants were incubated on CIM for 7 days. Whole leaf explants with developing calli were harvested for total RNA isolation. Transcript accumulation was analyzed by RT-PCR. The TUB gene was used as an internal control.
